# Handgrip strength in older adults and driving aptitude

**DOI:** 10.1590/0034-7167-2021-0729

**Published:** 2022-11-28

**Authors:** Maria Helena Lenardt, Tânia Maria Lourenço, Susanne Elero Betiolli, Maria Angélica Binotto, Clarice Maria Sétlik, Márcia Marrocos Aristides Barbiero

**Affiliations:** I Universidade Federal do Paraná. Curitiba, Paraná, Brazil; II Universidade Estadual do Centro-Oeste. Irati, Paraná, Brazil

**Keywords:** Elderly, Automobile Driving, Automobile Driver Examination, Hand Strength, Cross-sectional Studies, Adulto Mayor, Conducción de Automóvil, Examen de Aptitud para la Conducción de Vehículos, Fuerza de la Mano, Estudios Transversales, Idoso, Condução de Veículo, Exame para Habilitação de Motoristas, Força da Mão, Estudos Transversais

## Abstract

**Objective:**

to analyze handgrip strength as a predictor of the inability to drive in older adults.

**Method:**

a cross-sectional study conducted in traffic clinics with 421 older adults in Curitiba-Paraná from January 2015 to December 2018. A sociodemographic and clinical questionnaire, handgrip strength test, and queries from the National Registry of Qualified Drivers form were applied.

**Results:**

Reduced handgrip strength was not a predictor of inaptitude for vehicular driving (p=0.649). The predictors of inaptitude were: low education (p=0.011), incomplete elementary education (p=0.027), and cognition (p=0.020).

**Conclusion:**

reduced handgrip strength was not shown to predict for loss of driving skills in older adults. Low education level and reduced cognition level are conditions that were shown to be predictors for loss of vehicular driving license.

## RESUMO

**Objetivo:**

analisar a força de preensão manual como preditora de inaptidão para condução veicular de idosos.

**Método:**

estudo transversal realizado em clínicas de trânsito com 421 idosos em Curitiba-Paraná de janeiro de 2015 a dezembro de 2018. Aplicaram-se questionários sociodemográfico e clínico, teste de força preensão manual e consultas ao formulário de Registro Nacional de Condutores Habilitados.

**Resultados:**

A força de preensão manual reduzida não se mostrou preditora de inaptidão para a direção veicular (*p*=0,649). Os preditores de inaptidão foram: baixa escolaridade (*p*=0,011), ensino primário incompleto (*p*=0,027) e cognição (*p*=0,020).

**Conclusão:**

a força de preensão manual reduzida não se mostrou preditora para perda da habilitação na condução veicular de idosos. O baixo nível de escolaridade, o nível de cognição reduzida, são condições que se mostraram preditoras para a perda da habilitação na condução veicular.

## RESUMEN

**Objetivo:**

analizar la fuerza de prensión manual como factor predictivo de inaptitud para la conducción vehicular de adultos mayores.

**Método:**

se trata de un estudio transversal llevado a cabo en clínicas de tránsito de Curitiba, Paraná, entre 421 adultos mayores, de enero de 2015 a diciembre de 2018. Se aplicaron los cuestionarios sociodemográfico y clínico, la prueba de fuerza de prensión manual y se consultó el formulario del Registro Nacional de Conductores Habilitados.

**Resultados:**

la fuerza de prensión manual reducida no fue un factor predictivo de la incapacidad para conducir (*p*=0,649). Los predictores de inaptitud fueron: baja educación (*p*=0,011), estudios primarios incompletos (*p*=0,027) y cognición (*p*=0,020).

**Conclusión:**

la fuerza de prensión manual reducida no demostró ser predictora de la pérdida de habilidades de conducción vehicular en adultos mayores. El nivel de escolaridad bajo y el nivel cognitivo reducido despuntaron como predictores de la pérdida de habilitación en la conducción vehicular.

## INTRODUCTION

The increase in the number of senior drivers who use their vehicles as transportation for daily activities, work and/or leisure has become one of the challenges for society. This growth has caused changes in the traffic department statistics, especially regarding the age range of active drivers^(1)^.

According to data from the National Federation of Traffic Department Associations^(1)^, 3.6 million drivers over 60 were registered in Brazil in 2012. Updated data points to the state of Paraná with 1,874,511 registered drivers over 50 years old with a national driver’s license. In Curitiba, in 2020, 1,695 new driver’s licenses were issued to individuals within this age group^(2)^.

Driving a vehicle is a complex task that involves several abilities, such as cognitive, visual and motor skills, which present age-related changes, even when accompanied by a healthy aging process^(3)^. These changes include the age-related decline that affects neuromuscular and musculoskeletal function, with reduced muscle strength and decreased coordination and motor control^(4)^.

According to the criteria of the National Transit Council (CONTRAN), the muscular strength evaluation for obtaining a National Driver’s License (NDL) is performed in the neurological evaluation through the measurement of the Handgrip Strength (HGS). The value assigned for the candidate’s approval for the first driving license or vehicle renewal, established by the code of the National Traffic Council - Resolution No. 425 of November 27, 2012, is 20 kgf for women and 30 kgf for men^(5)^. These stipulated values for HGS are used by traffic clinics in attending the general population, i.e., from the young age group (18 years old) to older adults in advanced age for driver’s license approval.

HGS can identify muscle weakness in older adults and somehow interfere with the motor skills of older adults driver when driving, by decreasing strength and range of motion^(6-7)^. According to the Traffic Department, strength is an important requirement for the individual to maintain driving even in cars that use Anti-lock Braking System (ABS) brakes and power steering, maintaining trunk stability, endurance and coordination of movements^(8)^.

In the national and international scientific literature there are few studies that deal with the HGS, specifically regarding its reach and capacity to determine the muscle strength of older adult’s vehicle drivers. However, the HGS is a measure that can be measured in the most different scenarios of care for older adults’ population by all healthcare professionals, primarily by gerontologists, since it must be considered an essential part of the evaluation for the care of older adults with muscle loss.

We hope that the results of this study will provide evidence of the importance of the HGS marker for the evaluations of older adult’s applying for a CNH. Thus, we hope that the marker’s significance will contribute to adjusting these evaluations according to the specificity of older adults’ segment and, consequently, to make traffic safer. Considering the high number of older adults’ who seek clinics to renew their driver’s licenses, we can expect that this is a new action field to be sought by professionals who work with older adults’ segment.

## OBJECTIVE

To analyze handgrip strength as a predictor of driving aptitude in older adults.

## METHODS

### Ethical Aspects

The study followed the ethical principles of voluntary and consented participation by signing the Informed Consent Form (ICF) of each study participant, according to the recommendations in Resolution No. 466 /2012 of the National Health Council. The Research Ethics Committee evaluated this research project with the institution’s Human Subjects.

### Study design, setting and period

Quantitative, cross-sectional study guided by the Strengthening the Reporting of Observational Studies in Epidemiology (STROBE) tool, conducted in traffic clinics accredited to conduct physical and mental fitness tests for driving in Curitiba/PR. The study was developed in the period from January 2015 to December 2018.

### Study Participants

The study participants were older adults submitted to the physical and mental fitness test for the vehicular driving license exam. To calculate the sample size, we considered the city’s older adults’ population, estimated by the Brazilian Institute of Geography and Statistics based on the last census of 2010, which was 198,089 older adults. We considered a 95% confidence interval (95%CI), a significance level of 5%, a proportion estimate of 50%, and a sampling error of 5%. The sample’s final value was approximately 384 older adults, and 10% was added for the losses and refusals possibilities, resulting in a final sample of 421 older adults.

Inclusion criteria for the study included being 60 years old or older and scheduled to take the physical and mental fitness tests for a driving license in one of the traffic clinics. The exclusion criterion was that older adults had temporary physical limitations when taking the physical tests. We recruited 465 older adults to participate in the study. Among these, 44 refused to participate for the following reasons: 28 claimed lacks time, 11 reported a lack of interest, three did not agree to provide personal data, and two were dissatisfied with the final result of the physical and mental fitness test for a driving license. The final sample comprised 421 older adults.

The traffic clinics were selected by simple random sampling from an updated list (containing all 54 accredited clinics) made available by the Traffic Executive Body.

Distribution and scheduling of older adults for physical and mental fitness tests in traffic clinics are done by the Traffic Executive Body of Paraná. From this equitable, random, and impartial distribution of older adults in the clinics, we delimited an equal number of 35 older adults per clinic in order to guarantee the sample’s homogeneity.

Fourteen clinics located in different neighborhoods of the city were contacted. Two of them were excluded, one for not having adequate physical space to perform the tests and the other because the person in charge did not agree to participate in the research.

### Study protocol

Data collection occurred by applying structured questionnaires, a test to evaluate the HGS, and by consulting the National Registry of Qualified Drivers (RENACH) form.

As for the structured questionnaire, the sociodemographic and clinical characteristics of the sample were considered, represented by the interest variables: gender, age, marital status, education, monthly income, cognition level, disease number, history of falls in the last twelve months, reference to dizziness/vertigo/fainting, alcohol consumption, tobacco consumption, assistive technology use, medication use, and a record of hospital admissions.

For cognitive screening, the Mini-Mental State Examination (MMSE)^(9)^ was used, and for the cut-off points, the level of education was considered^(9)^, being 20 points for illiterates, 25 points for education between 1 and 4 years, 26.5 points for education between 5 and 8 years, 28 points for education between 9 and 11 years, and 29 points for individuals with more than 11 years of education.

As for the HGS evaluation, we used the criteria proposed by Linda Fried in her work on the Phenotype of Frailty^(10)^. To measure the HGS, a *Jamar®* hydraulic hand dynamometer in kilogram/force (Kgf) was used, following the recommendation of the American Society of Hand Therapists (ASHT)^(11)^. Older adults subjects were seated in a chair without armrests and with their feet on the floor, with adducted shoulder and elbow flexed at 90°, forearm in a neutral position, and wrist between 0 and 30° of extension. The test was performed with the dominant hand and repeated thrice, with a 60 second interval between measurements for rest^(12)^. The HGS was adjusted according to gender and body mass index, and the values that were in the lowest quintile were considered fragile for this marker^(10)^. For men, values below 30 kgf were considered low HGS, and for women, below 20 kgf^(5)^.^.^

In the RENACH consultation, the expert traffic examiner obtained and issued the final results of the physical and mental fitness exam for driving. The evaluation result considers the candidate fit, fit with restrictions, temporarily unfit, or unfit. For the “fit with restrictions” result, the specified needs are in the NDL’s registry Resolution nº425^(5)^, and the most demanded are: mandatory use of corrective lenses, mandatory use of hearing prosthesis, use of a vehicle with automatic transmission, mandatory use of steering wheel grip/handle/pommel, with a total of 23 restrictions that must be met to maintain the NDL.

### Data Analysis

The data were entered and coded in a *Microsoft Excel*® 2015 spreadsheet, and validation was performed by double-checking and checking the consistency of the information. The Stata/SE (version.14.1) computer program was used for statistical analysis. Quantitative variables were described by mean, standard deviation, and categorical by absolute and relative frequency. For the MMSE classification (good or bad) the points obtained in the test and education (years of study) were considered^(9)^. Handgrip strength was classified as reduced if <20 kgf for women and <30 kgf for men.

The chi-square and Fisher’s exact tests were used for the univariate factor analysis associated with inaptitude to drive. For the multivariate analysis, Logistic Regression models were adjusted considering impaired driving as the dependent variable (stepwise backward approach with a probability of 0.20 for removal of each variable), initially including the explanatory variables that showed p<0.25 in the univariate analysis and handgrip strength as the main variable of interest in the study. After fitting the models, the Wald test was used to assess the significance of each variable. The estimated measure of association was the odds ratio (OR) for which the 95% confidence intervals were presented. The normality condition of continuous quantitative variables was assessed by the *Kolmogorov-Smirnov* test. Values of p<0.05 indicated statistical significance.

## RESULTS

In 421 individuals, the mean age was 67.9, with a standard deviation of 6.7 years. Men were predominant (69.8%), in the age range of 60 to 69.9 years (66.0%), with higher education (38.0%), married (70.3%), living with a spouse (70.3%) and income of 1.1 to 3 minimum wages (32.5%). As for the clinical characteristics, a significant portion of older adults reported health problems (59.1%), and was on medication (66.5%), however, a minority reported hospitalization in the last year (10.2%). There is a predominance of older adults who did not use assistive technologies (98.8%), low alcohol consumption (78.1%) and low tobacco consumption (90.0%). The reduced HGS reached a percentage of 20.0% of the sample. Men had a mean of 37.4 Kgf with a standard deviation of 7.2 Kgf and women 25.1 Kgf with a standard deviation of 6.0 Kgf (Table 1).

**Table 1:** Predominance of sociodemographic and clinical characteristics and handgrip strength of older adults in driving, Curitiba, Paraná, Brazil, 2018 (N=421)

Variables	n (%)
Age (years)	
60 a 69	278 (66.0)
70 a 70	116 (27.6)
80 or more	27 (6.4)
Male gender	294 (69.8)
Marital status married/with partner	296 (70.3)
Education	
Incomplete primary education	89(21.2)
Elementary School Complete/High School Complete/Incomplete	172(40.8)
Higher education complete/incomplete	160(38.0)
Monthly income (minimum wage^*^)	
Up to 1	60(14.2)
1,1 a 3	137 (32.5)
3,1 or more	224 (53.3)
Disease Record	295(70.1)
Medication Use	280(66.5)
Reported falls in the last year	39(9.3)
No dizziness/fainting/vertigo	409(97.1)
Do not report alcohol consumption	329(78.1)
No tobacco use reported	379 (90.0)
Do not use assistive technologies	416(98.8)
No hospitalizations in the last year	378(89.8)
MMSE score with poor result ^**^	131 (31.1)
Reduced handgrip strength	84(20.0)

* National minimum wage in effect at the time of data collection R$ 954,00;

** MMSE - Mini-Mental State Examination.

In the univariate factor analysis associated with the results of the physical and mental fitness tests for driving by Detran (fit or unfit), the sociodemographic and clinical variables and the handgrip strength performed at Detran were evaluated (Table 2).

**Table 2:** Association between the results of the evaluation by Detran and the sociodemographic and clinical variables of older adults, Curitiba, Paraná, Brazil, 2018 (N=421)

Variables	Detran’s evaluation results				
	Fit / Fit with restriction		Unfit		*p* value
	n	%	n	%	
Age					0.622^‡^
60 to 69 years	260	93.5	18	6.5	
70 to 70 years	109	94	7	6.0	
80 years or more	24	88.9	3	11.1	
Gender					0.086 ^†^
Male	270	91.8	24	8.2	
Female	123	96.9	4	3.1	
Marital status					0.672 ^†^
Married/with partner	275	92.9	21	7.1	
Single/Divorced/Widowed	118	94.4	7	5.6	
Education					0.002^‡^
Incomplete primary education	99	86.8	15	13.2	
Elementary school complete/High school complete/incomplete	136	92.5	11	7.5	
Higher education complete/incomplete	158	98.8	2	1.3	
Monthly income					0.067^‡^
Up to one minimum wage	52	86.7	8	13.3	
1.1 to 3 minimum wages	128	93.4	9	6.6	
3.1 or more minimum wages	213	95.1	11	4.9	
Disease Record					0.524^†^
Yes	277	93.9	18	6.1	
No	116	92.1	10	7.9	
Medication Use					0.149^†^
Yes	265	94.6	15	5.4	
No	128	90.8	13	9.2	
Falls					0.314^†^
Yes	35	89.7	4	10.3	
No	358	93.7	24	6.3	
Dizziness/fainting/vertigo					0.567^†^
Yes	11	91.7	1	8.3	
No	382	93.4	27	6.6	
Alcohol Consumption					0.352^†^
Yes	84	91.3	8	8.7	
No	309	93.9	20	6.1	
Tobacco consumption					1^†^
Yes	40	95.2	2	4.8	
No	353	93.1	26	6.9	
Use of assistive technologies					1^†^
Yes	5	100.0	0	0.0	
No	388	93.3	28	6.7	
Hospitalization in the last year					0.053^†^
None	356	94.2	22	5.8	
1 or 2 hospitalizations	37	86.0	6	14.0	
MMSE^*^ score					0.002^†^
Good	278	70.7	12	42.9	
Bad	115	29.3	16	57.1	
Handgrip strength					0.468
Preserved	316	93.8	21	6.2	
Reduced	77	91.6	7	8.3	

* *DETRAN - Paraná’s Traffic Department; MMSE - Mini Mental State Examination; †Fisher's Exact Test (p ≤ 0,05) for the variables MMSE and Hospitalization in the last year; ‡Chi-square test (p ≤ 0,05) for variable education.*

For the multivariate model, handgrip strength and the variables p<0.25 in the univariate analysis (gender, education, hospitalization in the last year, MMSE score, monthly income, and medication use) were initially included. The variables that remained in the final model are shown in Table 3, Model 1. Additionally, a model was adjusted, keeping handgrip strength as the explanatory variable (Model 2 in Table 3). The result indicates that, adjusting for the other variables included in the model, there is no significant association between HGS and vehicular impairment in older adults (p=0.670, OR=1.24; 95% CI:0.46 - 3.30).

**Table 3:** Logistic regression model for variable handgrip strength associated with the results of the vehicle fitness tests of older adults, Curitiba, Paraná, Brazil, 2018

Variable	Rating	*p* ^*^	OR	IC95%
Model 1				
MMSE	Good			
	Bad	0.010	2.90	1.29 - 6.53
Last year's hospitalizations	None (ref)			
	1 or 2	0.051	2.76	1.00 - 7.63
Education	Higher Ed. inc/compl (ref)			
	Elementary School complete/High School inc/complete	0.016	6.60	1.42 - 30.6
	Up to Elementary School incomplete	0.002	10.4	2.31 - 47.2
Model 2				
Handgrip strength	Preserved (ref)			
	Reduced	0.670	1.24	0.46 - 3.30
MMSE	Good			
	Bad	0.009	2.95	1.31 - 6.66
Last year's hospitalizations	None (ref)			
Education	1 or 2	0.081	2.58	0.89 - 7.48
Escolaridade	Higher Ed. inc/compl (ref)			
	Elementary School complete/High School inc/complete	0.016	6.62	1.43 - 30.7
	Up to Elementary School incomplete	0.002	10.5	2.32 - 47.4

* *Logistic Regression Model and Wald test, p<0,05. HGS - Handgrip strength; MMSE - Mini-Mental State Examination; CI - Confidence Interval; ref - reference; inc - incomplete; compl - complete; OR - ODDS RATIO*

Although the predictive power of reduced HGS was not observed (OR: 1.24; 95%CI: 0.46 - 3.30) for older adults’ inability, we highlight that schooling has predictive power for this enabling outcome. Older adults with low schooling, represented by complete elementary school (p=0.016) and incomplete elementary school (p=0.002), when compared with higher education, presented approximately 7 and 10 times more chances of being considered unfit to drive, respectively (OR: 6.60; 95% CI: 1.42 - 30.6 and OR: 10.4; 95% CI: 2.31 - 47.2) (Table 3).

Similarly, Model 2 highlights the predictive power of cognition assessed by MMSE for the inability to drive a car (p=0.009), older adults identified with poor MMSE were three times more likely to be considered unfit for driving when compared to older adults with preserved cognition identified by MMSE (OR: 2.95; 95%CI: 1.31 - 6.66). Hospitalization in the last year was not a predictor of unfitness to drive.

## DISCUSSION

In the present study, there was no significant association between reduced PMF, and the physical and mental fitness test results of older adults performed by the traffic clinics (p=0.670). Of the 393 older adults people classified as fit or fit with restrictions, 77 of them (18.3%) had reduced PMF; however, this reduction did not make it impossible to obtain a license to drive a vehicle. Regardless of whether the car has an automatic or manual transmission, many maneuvers require strength in the hands (agility on the steering wheel) and lower limbs (braking), which are facilitated by muscle strength in these individuals.

Aging is a heterogeneous process that affects all individuals in different ways and to different degrees. However, the changes caused by aging often influence the ability of older adults to drive over time. Personalized intervention plans will increase the chances of older adults remaining independent, autonomous, and driving vehicles safely for longer^(13)^. The growing aging of the population and the increase in the number of older adults’ drivers must be considered by the spheres that perform the aptitude tests and authorize the permanence of older adults in driving vehicles.

Reduced HGS did not show predictive power to inaptitude to drive (p=0.670; OR=1.24; 95%CI: 0.46 - 3.30). Although studies point to the importance of identifying the loss of muscle strength (13-16), the reduced PMF did not influence the final result issued by the Traffic Department evaluators and the acquisition of the National Driver’s License (NDL) by older adults.

The Korean study, with 716 older adults people over 65 years of age, concluded that the chances of stopping driving decreased with each unit (kg) of handgrip strength (OR=0.939, 95% CI: 0.911 - 0.967; p<0.001)^(17)^. Researchers state that physical function is highly related to the interruption of driving, and HGS indicates the physical performance of older adults^(18)^.

Older adults who presented reduced HGS are believed to be in the initial phase of muscle strength loss; however, the decline is insufficient to make them unfit to drive a vehicle. We know that the decline in grip strength can be considered a strong predictor and marker of muscle weakness that may affect older adults over time, regardless of age, nutritional status, pre-existing diseases, lifestyle, inflammatory status, and mental status^(19-20)^.

The educational level and cognitive ability variables, compared to the HGS, were shown to have the greatest potential to predict the inability to drive. Low education is considered a risk for older adults’ population, considering the difficulty in receiving directions and interpreting the information provided along their route.

In the present study, the education level was considerably higher compared to studies developed with older adults individuals from other contexts, such as: community^(21)^, long-stay institution^(22)^ and hospitals^(23)^. We observed that older adults with incomplete elementary education (OR=10.5; 95%CI: 2.32 - 47.4) and complete elementary education, complete/incomplete high school (OR=6.62; 95%CI: 1.43 - 30.7) have a higher chance of being considered unfit to drive, when compared to older adults who had higher education. Education exerts a protective effect on cognition due to increased synaptic density in the neocortex, favoring neurogenesis and synaptogenesis at all stages of life^(24)^.

This is corroborated by an American study of 17,349 older adults (over 65 years of age), which aimed to identify social, psychological, and biomedical risk factors for the current and future driving cessation in older adults. In multivariate logistic regression to identify risk factors for older adults, the odds of current and future driving cessation were increased by 8% and 5% for each unit increase in education. The data reinforce that the higher the level of education of older adults, the longer they will be able to drive a vehicle^(25)^.

Cognitive ability is an element that must be considered when evaluating safe driving. The cognitive processes involved in driving require a good performance of executive function, memory, attention, and information processing speed. These cognitive mechanisms related to age can prevent the continuity of older adults’ driving activity^(26)^.

In Brazil, a study that determined the extent to which age, muscle strength, cognition, and balance are associated with braking performance in 62 middle-aged and 102 older adults^(4)^ showed that the variance in braking time was explained by 14% (p≤0.001) by cognitive change. The observed results indicated that changes related to age, physical function, and cognition could significantly interfere with the ability to perform critical vehicular driving tasks.

The variable hospitalization in the last year did not show statistical significance in predicting unfitness in regression Model 2, although it presents a statistical trend (p=0.051). Older adults under this condition showed a higher chance of unfitness for driving, considering that those hospitalized had 2.58 more chances (95%CI:0.89 - 7.48) to present this outcome. Aging and hospitalizations are considered risk factors for both loss of HGS and can also determine changes in cognition, affecting older adults driving continuity^(4)^.

The present study results are disturbing compared to the results of the traffic exams since some older adults considered fit to drive, upon evaluation by the clinics, do not have the necessary HGS for safe vehicular driving. The reduction in HGS cannot be underestimated since older adults need to have a minimum amount of strength to perform the basic tasks of driving a vehicle.

### Study limitations

Among the main limitations of the present study are the cross-sectional design, which does not allow the observation of the cause/effect relationship between the variables of interest. Another study limitation is the HGS assessment by the traffic clinics, which do not have precise instruments, in disagreement with what is recommended by the American Society of Hand Therapists^(11)^. The dynamometer used by the clinics provided divergent values, which made it difficult to compare those obtained with the instrument used in the present study. Also, the scarcity of studies in the scientific literature related to the theme of Handgrip Strength and driving in older adults is highlighted, which limited the discussions.

### Contributions to the field of gerontological nursing

For gerontological nursing, investigating the reduced PMF of older adults in other contexts, such as traffic clinics, means recognizing other yet unexplored fields of professional action. The early recognition of variables that interfere in the muscle decline of older adults’ drivers allows for recommending gerontological care to older adults drivers, those that prevent negative outcomes for muscle loss, in order to keep these older adults independent and autonomous for a longer time.

Gerontological nursing also recommends cognitive assessment in the process of the driver’s license examination for older adults. Identifying cognitive impairment should be part of the evaluations that older adults undergo to contribute to the effectiveness of these evaluations and, consequently, safer traffic for society, especially for older adults.

## CONCLUSION

Reduced handgrip strength was not a predictor of the inability to drive a vehicle in older adults’ drivers. Low educational level and reduced cognition level are predictors for losing a driving license, regardless of whether older adults had reduced HGS.

## SUPPLEMENTARY MATERIAL

Article extracted from the doctoral thesis “Força de preensão manual: marcador de fragilidade física em idosos submetidos ao exame de aptidão para habilitação veicular”, presented to the Universidade Federal do Paraná, Post-Graduation Program in Nursing, Curitiba, PR, Brazil.

## References

[1] Federação Nacional das Associações de Detran (2013). Segurança no trânsito para a terceira idade [Internet].

[2] Departamento de Trânsito do Paraná (DETRAN) (2019). Anuário Estatístico 2020 [Internet].

[3] Karthaus M, Falkenstein M (2016). Functional Changes and Driving Performance in Older Drivers: Assessment and Interventions. Geriatrics.

[4] Alonso AC, Peterson MD, Busse AL, Jacob-Filho W, Borges MTA, Serra MM (2016). Muscle strength, postural balance, and cognition are associated with braking time during driving in older adults. Exp Gerontol.

[5] Conselho Nacional de Trânsito (CONTRAN) (2012). Resolução nº 425, de 27 de novembro de 2012. Dispõe sobre o exame de aptidão física e mental, a avaliação psicológica e o credenciamento das entidades públicas e privadas de que tratam o art. 147, I e §§ 1º a 4º e o art. 148 do Código de Trânsito Brasileiro [Internet].

[6] Steiber N (2016). Strong or Weak Handgrip? normative reference values for the German Population across the life course stratified by sex, age, and body height. PloS ONE.

[7] Alley DE, Shardell MD, Peters KW, McLean RR, Dan TTL, Kenny AM (2014). Grip strength cutpoints for the identification of clinically relevant weakness. J Gerontol A Biol Sci Med Sci.

[8] Departamento Estadual de Trânsito do Estado do Paraná (DETRAN/PR) Portaria nº 303/2015-DG, de 03 de Junho de 2015.

[9] Brucki SMD, Nitrino R, Caramelli P, Berttolucci P HF, Okamoto IH (2003). Suggestions for utilization of the mini-mental state examination in Brazil. Arqui Neuropsiquiatr.

[10] Fried L, Tangen CM, Walston J, Newman AB, Hirsch C, Gottdiener J (2001). Frailty in older adults: evidence for a phenotype. J Gerontol A Biol Sci Med.

[11] Fess EE, Casanova JS (1992). Clinical assessment recommendations.

[12] Figueiredo IM, Sampaio RF, Mancini MC, Silva FCM, Souza MAPP (2017). Teste de força de preensão utilizando o dinamômetro Jamar. Acta Fisiátr.

[13] Cesari M, Calvani R, Marzetti E (2017). Frailty in older person. Clin Geriat Med.

[14] Morley JE (2016). Frailty and sarcopenia: the new geriatric giants. Rev Invest Clin [Internet].

[15] Cruz-Jentoft AJ, Baeyens JP, Bauer JM, Boirie Y, Cederholm T, Landi F (2010). Sarcopenia European consensus on definition and diagnosis: report of the European Working Group on Sarcopenia in older people. Age Ageing.

[16] Bohannon RW, Schaubert KL (2005). Test-retest reliability of grip-strength measures obtained over a 12-week interval from community-dwelling elders. J Hand Therapy.

[17] Hwang Y, Ryung G (2018). Predictors of driving cessation in community-dwelling older adults: a 3-year longitudinal study. Transport Res Part F Traffic Psychol Behav.

[18] Legrand D, Adriaensen W, Vaes B, Mathei C, Wallemacq P, Degryse J (2013). The relationship between grip strength and muscle mass (MM), inflammatory biomarkers and physical performance in community-dwelling very old persons. Arc Geront Geriat.

[19] Xue Q, Walston JD, Fried LP, Beamer BA (2011). Prediction of risk of falling, physical disability, and frailty by rate of decline in grip strength: the Women’s Health and Aging Study. Arch Intern Med.

[20] Bohannon R (2019). Minimal clinically important difference for grip strength: a systematic review. J Phys Ther Sci.

[21] Brigola AG, Ottaviani AC, Souza EN, Rossetti ES, Terassi M, Oliveira NA (2018). Descriptive data in different paper-based cognitive assessments in elderly from the community Stratification by age and education. Dement Neuropsychol.

[22] Matos AIP, Mourão I, Coelho E (2016). Interação entre a idade, escolaridade, tempo de institucionalização e exercício físico na função cognitiva e depressão em idosos. Motriz.

[23] Cafagna G, Seghieri C (2017). Educational level and 30-day outcomes after hospitalization for acute myocardial infarction in Italy. BMC Health Services Research.

[24] Brucki SMD, Nitrini R (2010). Mini-Mental State Examination among lower educational levels and illiterates: Transcultural evaluation. Dement Neuropsychol.

[25] Dugan E, Lee CM (2013). Biopsychosocial risk factors for driving cessation: findings from the health and retirement study. J Aging Health.

[26] Miller SM, Taylor-Pilie RE, Insel KC (2016). The association of physical activity, cognitive processes and automobile driving ability in older adults: a review of the literature. Geriatr Nurs.

